# Direct Probing of Trap Dynamics in *β*‐Ga_2_O_3_ Schottky Barrier Diodes Using Single‐Voltage‐Pulse Characterization

**DOI:** 10.1002/advs.202518859

**Published:** 2025-12-14

**Authors:** Thanh Huong Vo, Sunjae Kim, Ji‐Hyeon Park, Dae‐Woo Jeon, Wan Sik Hwang, Jinyoung Hwang

**Affiliations:** ^1^ Department of Materials Science and Engineering Korea Aerospace University Goyang 10540 Republic of Korea; ^2^ Department of Smart Air Mobility Korea Aerospace University Goyang 10540 Republic of Korea; ^3^ Korea Institute of Ceramic Engineering and Technology Jinju 52851 Republic of Korea; ^4^ Department of Electrical and Electronic Engineering Korea Aerospace University Goyang 10540 Republic of Korea

**Keywords:** carrier trapping, single‐voltage‐pulse characterization, transient current analysis, trap states, *β*‐Ga2O3 Schottky barrier diodes

## Abstract

Gallium oxide (*β*‐Ga_2_O_3_) is a promising ultrawide‐bandgap semiconductor for next‐generation power electronics, but its performance is strongly limited by trap states that capture carriers. In this study, a single‐pulse characterization method is presented to directly probe trap dynamics in *β*‐Ga_2_O_3_ Schottky barrier diodes (SBDs). Transient current responses are systematically investigated under varying pulse widths, rise and fall times, amplitudes, and temperatures. The results reveal that traps in the neutral region progressively participate in electron capture, resulting in current decay during the constant‐voltage phase. Additionally, a delayed trap response produces asymmetry between the ramp‐up and ramp‐down transients. Analysis of the current decay yielded a trap density of ≈5×10^14^ cm^−2^, representing the total trap density near the Schottky junction. Exponential fitting provides a carrier capture time constant of ≈ 30 µs at a forward bias of 2 V, consistent with the onset of trap‐induced current degradation. Temperature‐dependent measurements indicate that carrier capture is suppressed at elevated temperatures, resulting in a trap activation energy of ≈0.16 eV. These findings demonstrate that the single‐pulse method offers a straightforward and effective approach for evaluating trap states under practical operating conditions in *β*‐Ga_2_O_3_ devices.

## Graphical abstract

A single‐pulse approach uncovers trap dynamics in *β*‐Ga_2_O_3_ Schottky barrier diodes. Transient current profiling reveals rapid electron capture, delayed trap filling, and clear thermal effects. Extracted trap densities, capture time constants, and activation energies indicate that this simple pulse technique enables effective evaluation of trap states under realistic device operation conditions.

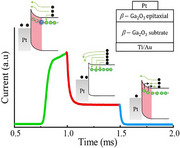

## 1 Introduction

Gallium oxide (*β*‐Ga_2_O_3_) is a semiconductor material with an ultrawide bandgap (≈4.8 eV) and an exceptionally high theoretical breakdown electric field (≈8 MV cm^−1^).^[^ ^1^, ^2^ ^]^ These unique properties make it a promising candidate for next‐generation power electronics, solar‐blind ultraviolet photodetectors, and devices designed for harsh environments.^[^ ^3^, ^4^, ^5^, ^6^, ^7^ ^]^ However, the presence of defect states within the bandgap often hinders the material from reaching its full potential.^[^ ^8^, ^9^, ^10^ ^]^ The origin of these defect states in *β*‐Ga_2_O_3_ is complex and generally attributed to both intrinsic and extrinsic sources.^[^ ^11^ ^]^ Intrinsic defects, which arise from imperfections in the crystal lattice, include native point defects such as gallium vacancies (V_Ga_), oxygen vacancies (V_O_), and their associated complexes.^[^ ^9^, ^12^ ^]^ Extrinsic defects, on the other hand, originate from the unintentional incorporation of impurity atoms, such as silicon (Si) and iron (Fe), during high‐temperature growth processes.^[^ ^12^, ^13^ ^]^ These defects introduce localized energy levels within the bandgap that act as traps, capturing and emitting charge carriers. These traps can significantly impact device operation and reliability, including current collapse, threshold voltage instability, and degradation of switching characteristics.^[^ ^14^, ^15^, ^16^, ^17^, ^18^ ^]^ Therefore, a precise characterization and comprehensive understanding of these trap states are crucial for further advancing *β*‐Ga_2_O_3_‐based device technologies.

Significant efforts have been devoted to identifying and quantifying trap states in *β*‐Ga_2_O_3_ through established analytical techniques, including Deep Level Transient Spectroscopy (DLTS) and Deep Level Optical Spectroscopy (DLOS).^[^ ^8^, ^19^, ^20^, ^21^ ^]^ These techniques have provided valuable insights into trap energy levels, capture cross‐sections, and trap concentrations.^[^ ^15^, ^22^, ^23^ ^]^ Capacitance‐voltage (C–V) characterization has also been employed to assess the spatial distribution of defects within device structures.^[^ ^2^, ^24^, ^25^ ^]^ Despite their effectiveness, these methods often require complex instrumentation, extensive temperature or frequency sweeps, and involve externally induced perturbations, such as electrical pulses or optical excitation.^[^ ^26^, ^27^, ^28^, ^29^ ^]^ Furthermore, charge pumping techniques, commonly used to investigate carrier populations and interface trap dynamics, are inherently tailored to three‐terminal structures, such as Metal‐Oxide‐Semiconductor Field‐Effect Transistors (MOSFETs).^[^ ^6^, ^30^, ^31^, ^32^ ^]^

This study introduces and validates a straightforward yet effective method that employs a single square voltage pulse to comprehensively analyze transient current responses in *β*‐Ga_2_O_3_ Schottky barrier diodes (SBDs). The application of these pulses establishes non‐equilibrium conditions, enabling direct observation of carrier injection, trapping, and subsequent emission dynamics, as manifested in the characteristic current transients. By carefully analyzing the initial current peak, subsequent decay, and turn‐off response, we quantify trap parameters, including the effective trapped state density, trap energy levels, and capture rates. The proposed single‐pulse technique is experimentally simple, susceptible to dynamic trapping phenomena, and directly relevant to the practical operating conditions of devices. This approach offers clear advantages over conventional methods by simplifying both device structure and measurement setup requirements, thereby laying the groundwork for improved understanding and control of trap‐related effects in *β*‐Ga_2_O_3_ devices through direct probing of carrier trapping phenomena.

## 2 Results and Discussion

**Figure 1:**
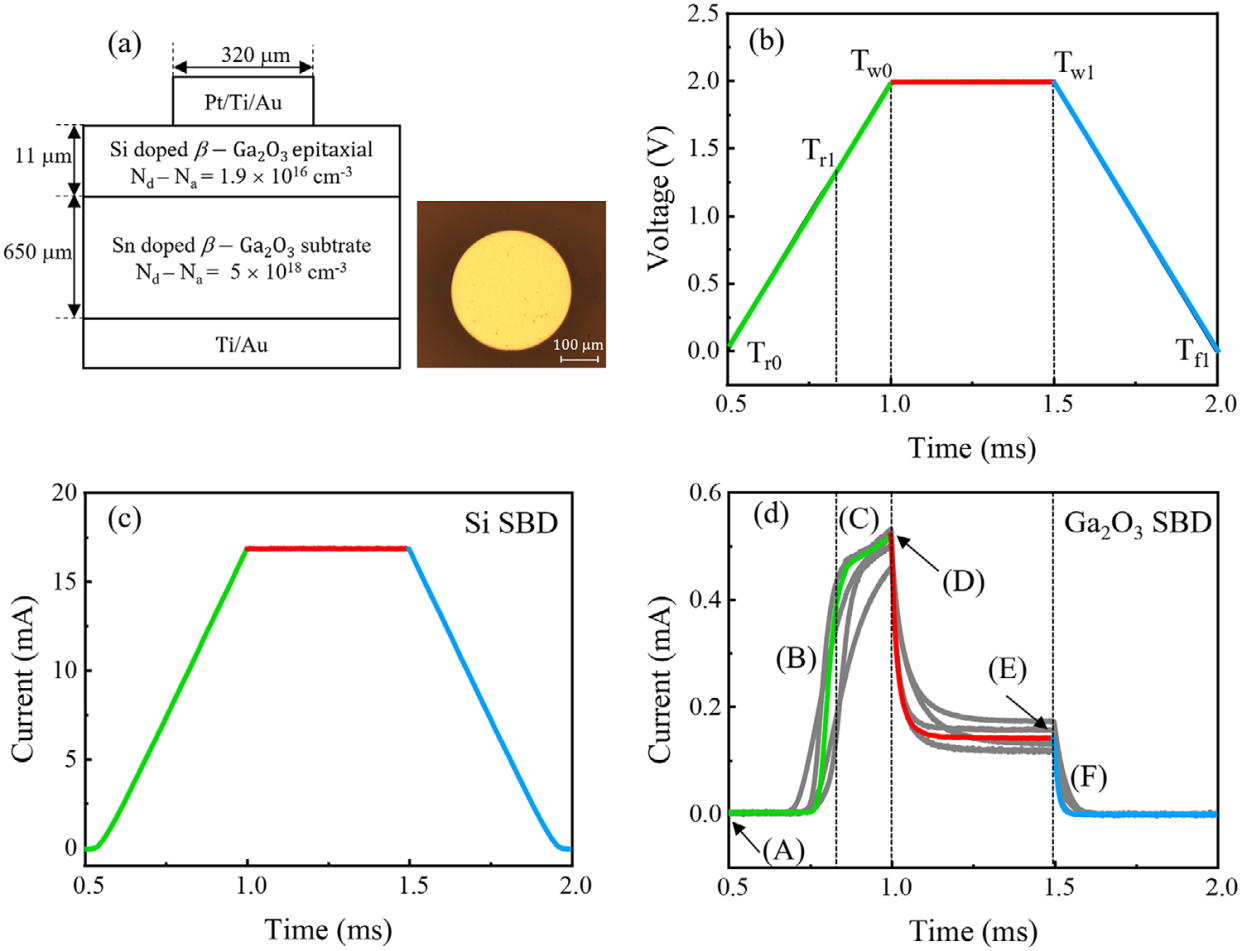
Transient current responses of SBDs under a single voltage pulse. a) Schematic cross‐section of the *β*‐Ga_2_O_3_ SBD structure and an optical microscope image of the fabricated circular device on the front side, with an effective device area of 8.04 × 10^−4^ cm^2^ (scale bar: 100 µm). b) A voltage pulse with an amplitude of 2 V, rising time (T_r_) of 0.5 ms, pulse width (T_w_) of 0.5 ms, and falling time (T_f_) of 0.5 ms. T_w0_ and T_w1_ denote the beginning and end of the constant‐voltage phase, while T_r0_ and T_f1_ mark the start of the rising edge and the end of the falling edge. T_r1_ indicates the point at which the slope of the rising current begins to decrease in the *β*‐Ga_2_O_3_ SBD. c) Transient current response of a commercial Si SBD. d) Transient current response of the *β*‐Ga_2_O_3_ SBD investigated in this study. Measurements were repeated on six devices, shown as the gray traces, and the colored solid lines represent the averaged response.

**Figure** **1**a illustrates a schematic cross‐sectional diagram of the *β*‐Ga_2_O_3_ SBD investigated in this study. The device comprises the Pt/Ti/Au metal stack formed on an 11 µm‐thick, n‐type *β*‐Ga_2_O_3_ epitaxial layer, which is grown on a 650 µm‐thick, n‐type *β*‐Ga_2_O_3_ substrate. A Ti/Au ohmic contact is deposited on the substrate backside. Figure 1b shows the voltage waveform applied during pulsed current (PC) measurements. A single square voltage pulse with an amplitude of 2 V was applied to drive the SBD from equilibrium to a forward bias condition. The voltage ramps linearly from 0 to 2 V over a rising time (T_r_) of 0.5 ms, starting at *t* = T_r0_ and reaching 2 V at time *t* = T_w0_. Afterward, the voltage is held constant at 2 V for a pulse width (T_w_) of 0.5 ms, extending from *t* = T_w0_ to *t* = T_w1_. Finally, the voltage decreases linearly from 2 to 0 V over a falling time (T_f_) of 0.5 ms, from *t* = T_w1_ to *t* = T_f1_.

Figure 1c shows the current transient response of a commercial Si SBD under the applied voltage pulse. The Si SBD exhibits near‐ideal behavior, with the current closely following the change in the applied voltage. The current increases to 17.5 mA during the voltage ramp‐up, remains constant throughout the pulse width, and then decreases during the voltage ramp‐down period. The current responses during the voltage ramp‐up and ramp‐down are symmetric. In contrast, Figure 1d presents the current transient response of the *β*‐Ga_2_O_3_ SBD, which displays distinct non‐ideal features. To ensure data reliability, measurements were performed on six devices, with the individual responses shown as gray traces, while the colored solid lines represent the averaged response.

During the voltage ramp‐up from 0 to 2 V, the current increases rapidly at first. However, once the voltage exceeds ≈1.4 V, the rate of current increase begins to decrease noticeably; this point is defined as *t* = T_r1_. As the voltage continues to rise, a pronounced current peak appears at the end of the rising phase. Upon entering the constant‐voltage phase at *t* = T_w0_, the current begins to decay and gradually saturates, in contrast to the stable response observed in the Si SBD. Similar transient current‐degradation phenomena have been reported in other semiconductor systems during constant‐voltage phases, with pulse widths ranging from sub‐millisecond to millisecond durations.^[^ ^33^, ^34^, ^35^ ^]^ Additionally, the current response during the voltage ramp‐down is asymmetric compared to the ramp‐up. Specifically, the current decreases more abruptly during the falling period. During the voltage ramp‐up, the current turns on at t ≈ 0.75 ms, corresponding to a forward bias of ≈1.03 V. In contrast, during the ramp‐down, turn‐off occurs at t ≈ 1.58 ms, when the voltage has decreased to ≈1.69 V.

**Figure 2:**
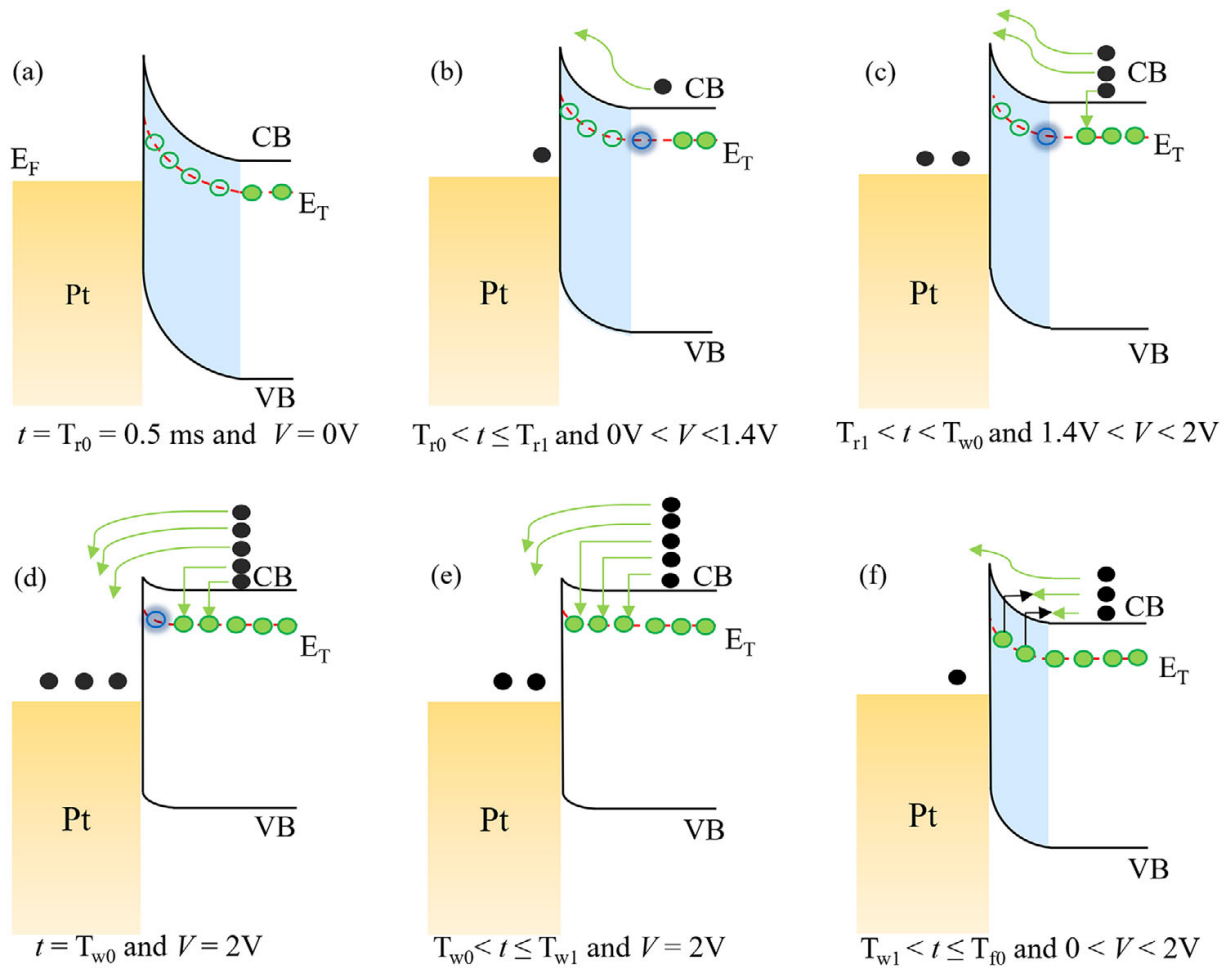
Energy band diagrams of the *β*‐Ga_2_O_3_ SBD at different stages of the voltage pulse, corresponding to labels (A) – (F) in Figure 1d, illustrating the evolution of trap‐assisted carrier dynamics. a) Equilibrium at *t* = T_r0_ and V = 0 V; b) Early forward‐bias ramp‐up phase (T_r0_ < t ≤ T_r1_); c) Intermediate ramp‐up phase (T_r1_ < t < T_w0_); d) Peak forward bias at V = 2 V and t *=* T_w0_; e) Constant‐voltage phase (T_w0_ < t ≤ T_w1_) when the current approaches saturation; f) Ramp‐down phase (T_w1_ < t < T_f1_). Black circles represent free electrons. Green‐filled circles denote traps that have captured electrons, while green empty circles represent unoccupied traps in the depletion region. Blue empty circles correspond to traps in the neutral region that have not yet captured carriers but may subsequently become active.

The transient current response of the *β*‐Ga_2_O_3_ SBD underscores the significant influence of trap states within the bandgap on carrier transport. To elucidate the physical mechanisms underlying the observed transient behavior in each voltage phase, **Figure** **2** illustrates the corresponding energy band diagrams. The labels (A) – (F) in Figure 1d correspond directly to the respective operating conditions illustrated in Figure 2. Under the equilibrium condition, as portrayed in Figure 2a, the traps within the depletion region, shaded in blue, remain mostly unoccupied because the built‐in electric field depletes free electrons. The empty green circles depict the vacant traps. In contrast, traps in the neutral region can capture carriers and become occupied, as represented by green‐filled circles.

During the voltage ramp‐up phase, corresponding to the segment labeled (B) in Figure 1d and spanning from t = T_r0_ to t = T_r1_, the energy barrier for electrons in the *β*‐Ga_2_O_3_ conduction band decreases, and the depletion region shrinks, resulting in forward‐bias current flow. During this period, the current increases sharply in response to the rising forward bias as electrons are injected from the *β*‐Ga_2_O_3_ into the metal across the Schottky barrier. Notably, the traps, which were previously empty and located within the depletion region at equilibrium, now reside in the neutral region due to the reduction in the width of the depletion region. However, at this early stage, the time is insufficient for significant trapping to occur, so these traps remain unoccupied. Blue empty circles in Figure 2b represent these unfilled traps in the neutral region.

After a certain amount of time passes, some of the electrons injected from *β*‐Ga_2_O_3_ into the metal start to be captured by the empty traps in the neutral region. As the energy band diagram for this period, shown in Figure 2c, indicates, traps capture some of the electrons flowing from *β*‐Ga_2_O_3_ to the metal, thereby preventing them from reaching the metal and resulting in current degradation. Therefore, the current increase rate cannot follow the forward bias increase, which corresponds to the current increasing rate degradation that occurs in the period labeled as (C) in Figure 1d, spanning from *t* = T_r1_ to T_w0_.

When the applied bias reaches 2 V at *t* = T_w0_, both the energy barrier and depletion width are minimized, resulting in the maximum forward current. This condition corresponds to the point labeled (D) in Figure 1d, with the corresponding energy band diagram shown in Figure 2d. At this moment, electron injection from the *β*‐Ga_2_O_3_ into the metal reaches its peak. Simultaneously, traps that have become located in the neutral region due to the reduced depletion width capture some of the electrons. However, not all traps participate in the capture process, as the time between their transition into the neutral region and the onset of peak bias is too short to initiate electron trapping. Blue empty circles in Figure 2d depict these unoccupied traps.

During the constant‐voltage phase at 2 V, the forward bias remains constant. Meanwhile, an increasing number of traps in the neutral region begin capturing injected electrons over time. It causes the current to gradually decrease until it stabilizes at a saturated level, indicating that all accessible traps have participated in the electron capture process. Figure 2e presents the energy band diagram corresponding to this condition. The magnitude of the observed current decay can be explained by the electrostatic effect of trapped charges. When the forward bias is applied, the depletion region narrows, and a high concentration of electrons is injected into the newly neutral region. As these electrons are captured by traps located at the advancing edge of the space charge region, they form a sheet of fixed negative charge. This localized charge distribution introduces an additional potential barrier through Coulomb repulsion, which opposes the transport of subsequent electrons toward the metal contact.^[^ ^35^, ^36^ ^]^

Finally, at *t* = T_w1_, the applied voltage begins to decrease from 2 to 0 V. This ramp‐down phase is labeled (F) in Figure 1d, with the corresponding energy band diagram shown in Figure 2f. During this period, the forward bias decreases, which increases both the energy barrier height and the depletion width at the Schottky junction, thereby reducing electron injection from the *β*‐Ga_2_O_3_ into the metal. Unlike the ramp‐up phase, however, some traps continue to capture electrons even after returning to the depletion region, owing to their slow response time. As a result, more electron trapping occurs at a given bias during the ramp‐down than during the ramp‐up, leading to a lower current under the same bias conditions. This behavior results in the asymmetric current transient response observed in Figure 1d.

**Figure 3:**
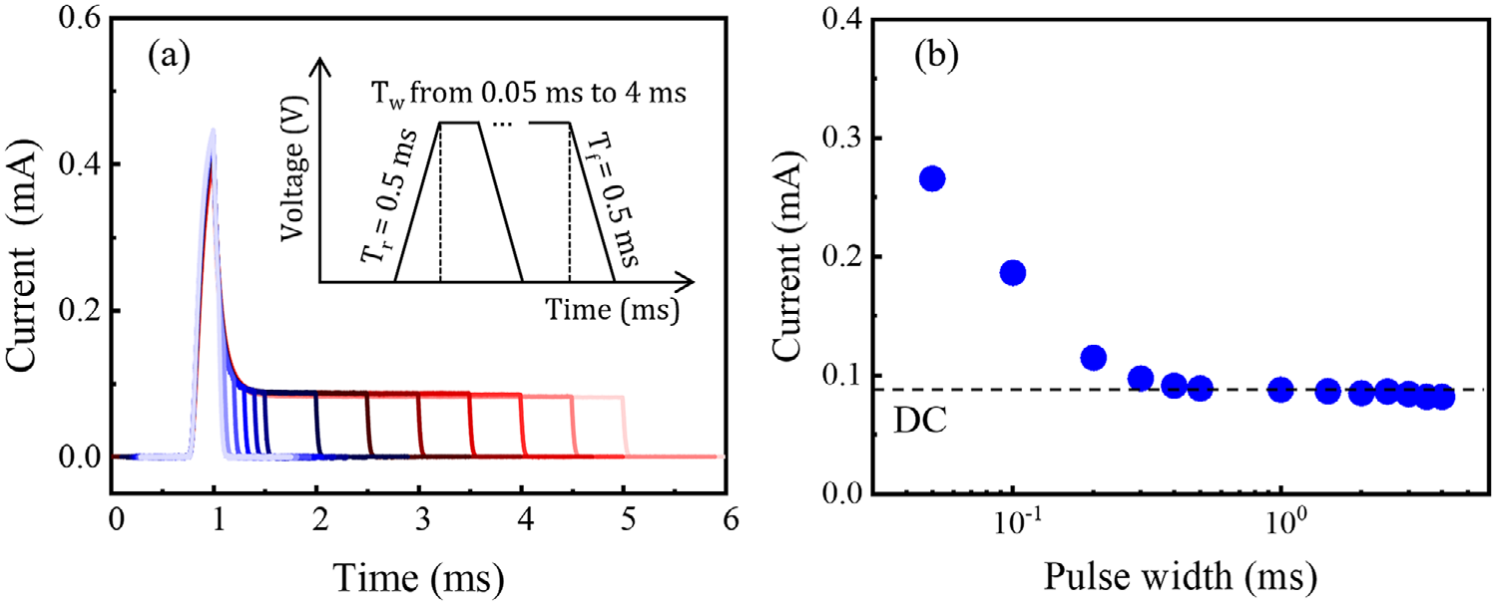
a) Transient current responses of the *β*‐Ga_2_O_3_ SBD under voltage pulses with T_w_ varied from 0.05 ms to 4 ms, while keeping T_r_ and T_f_ constant at 0.5 ms. The inset shows the applied voltage waveform illustrating the variation of T_w_. b) Current measured at the end of the constant‐voltage period (*t* = T_w1_) as a function of T_w_. The dashed line denotes the steady‐state DC current level at the forward bias of *V* = 2.0 V.

To further investigate the influence of trap states in the transient current response, pulse‐width‐dependent measurements were performed, as shown in **Figure** **3**. In these measurements, T_r_ and T_f_ were fixed at 0.5 ms, while T_w_ was varied from 0.05 ms to 4 ms, as illustrated in the inset of Figure 3a. The transient responses in Figure 3a reveal that the overall shape of the current decay during the constant‐voltage phase is independent of T_w_. Specifically, the current transients measured with shorter pulses precisely overlap with the initial portions of those measured with longer pulses. In the case of shorter T_w_, the constant‐voltage period ends before the current has sufficient time to stabilize, leading to an abrupt decrease in current as the voltage enters the ramp‐down phase.

Figure 3b quantifies the current measured at the end of the constant‐voltage phase (*t* = T_w1_) as a function of T_w_. The current at T_w1_ decreases with increasing T_w_ and ultimately saturates at the steady‐state DC level, indicated by the dashed line. During the voltage ramp‐up, the depletion width continuously decreases, causing traps initially located within the depletion region to become progressively located within the neutral region until *t* = T_w0_. At the onset of the constant‐voltage phase, steady state has not yet been reached, as the traps in the neutral region require additional time to complete electron capture. Consequently, for shorter T_w_, the available time is insufficient for complete trapping, which results in higher transient currents at *t* = T_w1_. In contrast, longer T_w_ allows all neutral region traps to participate fully, enabling the trapping process to reach a steady state and causing the transient current to converge toward the DC level.

**Figure 4:**
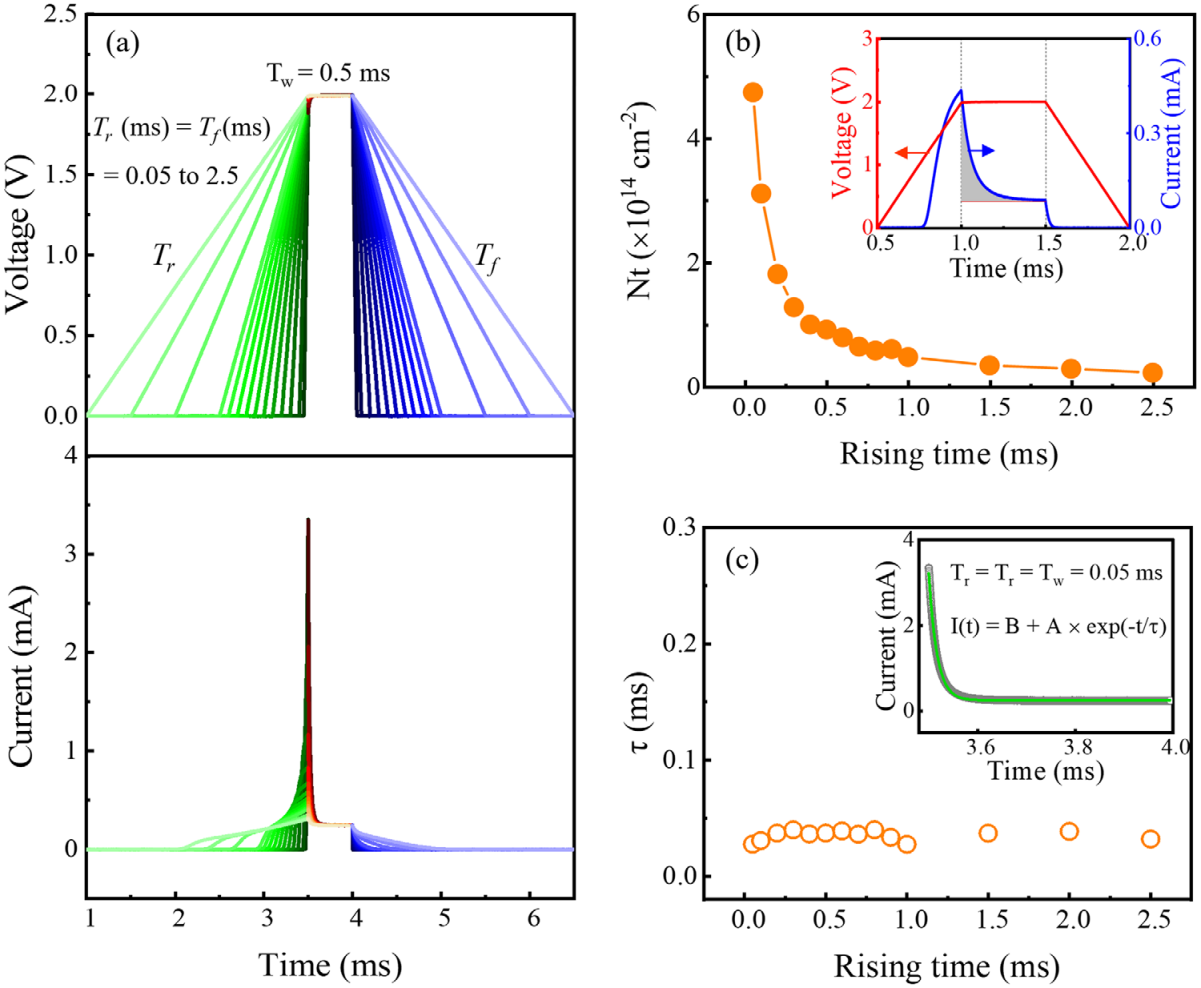
a) Applied voltage waveforms with varying T_r_ and T_f_ from 0.05 to 2.5 ms and the corresponding transient current responses of the *β*‐Ga_2_O_3_ SBD. b) Extracted trapped carrier density (N_t_) as a function of T_r_. The inset illustrates the shaded area under the transient current curve, which is used to calculate N_t_. c) Carrier capture time constant (τ) as a function of T_r_. The inset shows the exponential fitting of the transient current during the constant‐voltage phase, together with the fitting equation.

**Figure** **4**a presents applied voltage profiles with varying T_r_ and T_f_ from 0.05 to 2.5 ms, along with the corresponding transient current responses of the *β*‐Ga_2_O_3_ SBD. The pulse width (T_w_) and amplitude are fixed at 0.5 ms and 2.0 V, respectively. The choice of T_w_ = 0.5 ms corresponds to the condition at which the current reaches steady state during the constant‐voltage phase, as demonstrated previously in Figure 3b. A notable observation from the transient current response in Figure 4a is that the peak current value at the end of the ramp‐up phase (at *t* = T_w0_) increases significantly as T_r_ decreases. For instance, the peak current reaches ≈3.4 mA when T_r_ = 0.05 ms, compared to ≈0.3 mA when T_r_ = 2.5 ms.

**Figure 5:**
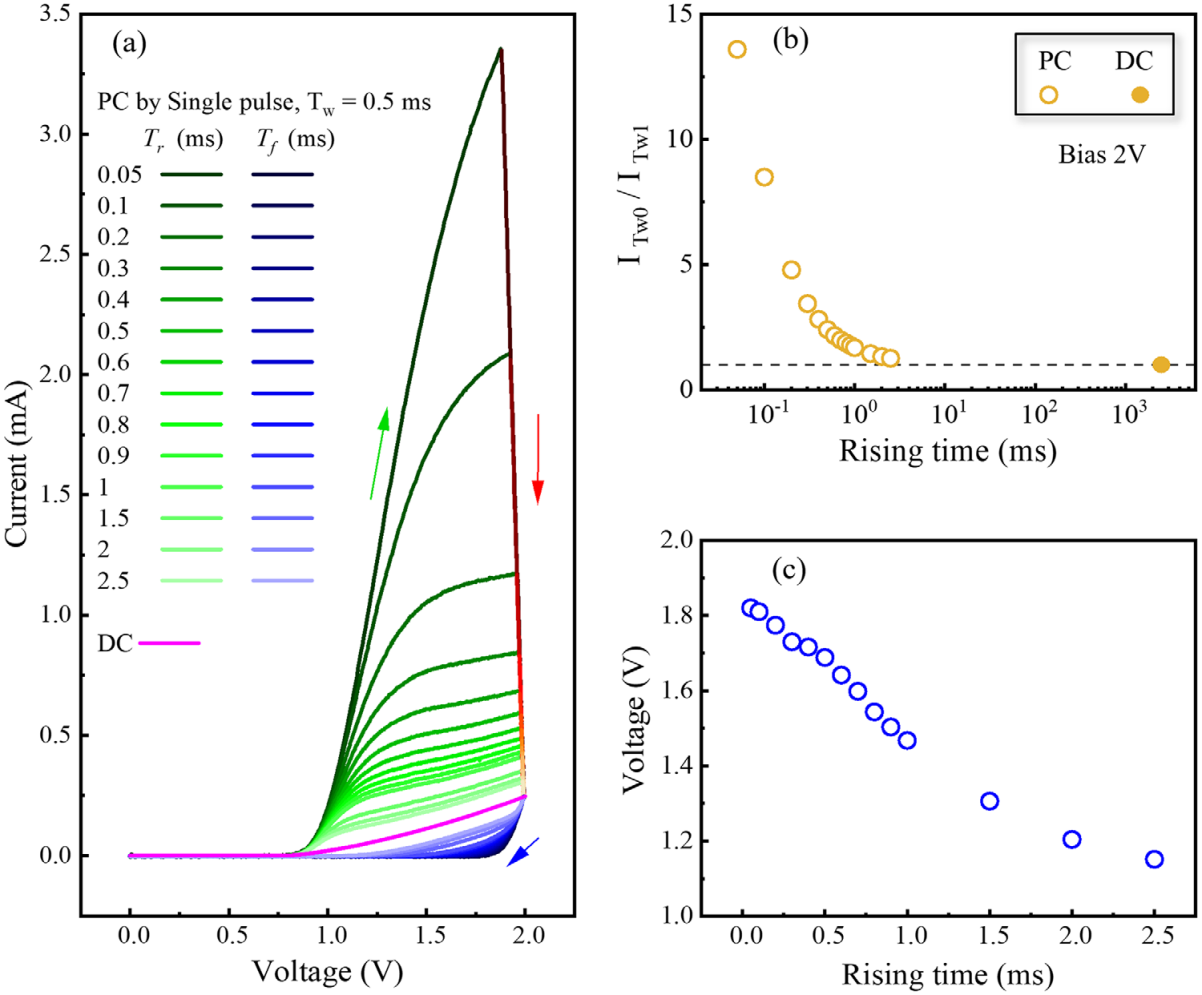
a) *I–V* characteristics extracted from the ramp‐up (green) and ramp‐down (blue) segments of the current transients in Figure 4a. The DC I–V curve is shown in magenta for comparison. b) Ratio of the peak current at *t* = T_w0_, I_Tw0_, to the current at the end of the constant‐voltage phase at *t* = T_w1_, I_Tw1_. The dashed line denotes 1, corresponding to the DC steady‐state condition. c) Forward bias at which the current decreases to 0.1 mA, the value at V = 0.75 V under the DC steady‐state condition, as a function of T_r_.

To more clearly visualize the transient behavior with varying T_r_ and T_f_, **Figure** **5**a shows the *I–V* characteristics derived from the ramp‐up and ramp‐down segments of the current transients in Figure 4a. The curves for the ramp‐up and ramp‐down segments are shown in green and blue, respectively. During ramp‐up, the current begins to rise at ≈V = 0.75 V for all T_r_ values, reflecting a consistent turn‐on behavior from the initial equilibrium state. For short rise times (e.g., T_r_ ≤ 0.05 ms), the current increases sharply with a consistently steep slope over the entire ramp‐up range. As T_r_ increases, however, the current rise evolves into a two‐stage behavior: an initial steep increase followed by a noticeably reduced slope before reaching the peak current.

This two‐stage rise reflects the interplay between rapid carrier injection, which occurs concurrently with the increasing forward bias, and delayed carrier capture by traps. For short T_r_, the ramp‐up phase ends before substantial electron capturing can occur, resulting in a high peak current dominated by carrier injection at V = 2.0 V. As T_r_ becomes comparable to or exceeds the characteristic time constant for electron capture, traps begin to capture carriers during the ramp‐up phase. Once trapping becomes prominent, the rate of current increase is substantially reduced. Consequently, longer T_r_ leads to more pronounced trapping effects during the voltage rise, thereby lowering the peak current reached at *t* = T_w0_. To quantitatively examine this trend, Figure 5b plots the ratio of the peak current at *t* = T_w0_, I_Tw0_, to the current at the end of the constant‐voltage phase at *t* = T_w1_, I_Tw1_. This ratio sharply decreases from ≈14 at T_r_ = 0.05 ms and eventually saturates at a value of 1, indicating that the transient current reaches steady state already at *t* = T_w0_.

Figure 5a also displays the *I–V* characteristics during the ramp‐down phase for different T_f_ values, revealing that the current returns to the off‐state level at higher forward bias as T_f_ decreases. For all T_f_ values, the ramp‐down phase begins from a steady‐state condition at *V* = 2.0 V, where the depletion width is minimized, and all available traps in the neutral region are actively engaged in carrier capture. As the forward bias decreases from 2.0 to 0 V, the depletion width expands, and carrier injection from *β*‐Ga_2_O_3_ to the metal reduces in immediate response to the voltage drop. However, traps that re‐enter the depletion region during this transition exhibit a delayed response to the changing bias, continuing to capture carriers even after shifting into the depletion region.

For very short T_r_, the applied forward bias returns to 0 V before the traps can respond to the voltage change, implying that traps that have re‐entered the depletion region still actively capture carriers. In this case, the sustained high rate of carrier capture keeps the current suppressed, so that even a slight reduction in carrier injection due to the decreasing forward bias is sufficient to drive the current to the off‐state level. As a result, the current reaches the off‐state level at a higher forward bias than under steady‐state conditions. Conversely, for longer T_r_, the gradual voltage change allows sufficient time for the carrier capture to subside during the ramp‐down phase. Therefore, a larger reduction in forward bias is required to sufficiently suppress carrier injection and bring the current to the off‐state level. Figure 5c quantifies the forward bias at which the current reaches 0.1 mA, the value corresponding to the current level at V = 0.75 V under DC conditions, as a function of T_r_. For T_r_ = 0.05 ms, this occurs at 1.85 V, whereas the required voltage decreases monotonically with increasing T_r_, reaching 1.0 V at T_r_ = 2.5 ms.

From the current decay observed during the constant‐voltage phase, which originates from carrier capture by traps near the Schottky barrier junction, the trapped carrier density, N_t_, can be estimated using the following expression:

(1) $$N_{t}=\frac{1}{\mathrm{qA}}\;\int_{T_{w0}}^{T_{w1}}\left\{I\left(t\right)-I_{\mathrm{Tw}1}\right\}\mathrm{dt}$$

where q is the electron charge, A is the diode area, I(t) is the transient current, and I_Tw1_ is the current at *t* = T_tw1_. The integral corresponds to the shaded gray area of the transient current curve shown in the blue curve in the inset of Figure 4b. As shown in Figure 4b, N_t_ decreases with increasing T_r_. This trend arises because longer rise times allow a larger fraction of traps to capture carriers during the ramp‐up phase, resulting in fewer carriers being captured in the constant‐voltage phase. When T_r_ is very short, on the order of 0.05 ms or less, carrier capture during the ramp‐up phase is negligible. Under the condition, the extracted N_t_, ≈4.75 × 10^14^ cm^−2^, represents the total trap density in the region near the Schottky barrier junction. This value is consistent with previously reported estimates of trap density in *β*‐Ga_2_O_3_ SBDs, confirming the reliability of the present analysis.^[^ ^37^ ^]^

Additionally, from the exponential fitting of the transient current during the constant‐voltage phase, the carrier capture time constant, τ, was extracted for different T_r_ values. The fitting function, shown in the inset of Figure 4a, is expressed as I(*t*) = B + A×exp(−*t*/τ), and it provides excellent agreement with the experimental curves. The extracted τ values remain nearly constant regardless of T_r_, with values close to 30 µs. This result is consistent with the behavior observed in Figure 5a, where significant carrier capture becomes apparent during the voltage ramp‐up phase when T_r_ exceeds 0.05 ms. Furthermore, reports using Deep‐Level Transient Spectroscopy (DLTS) on Ga_2_O_3_ have identified electron traps with capture time constants in the microsecond range (≈10 µs).^[^ ^38^ ^]^ This value is in good agreement with our experimental data, which enhances the reliability of our measurement technique.

**Figure 6:**
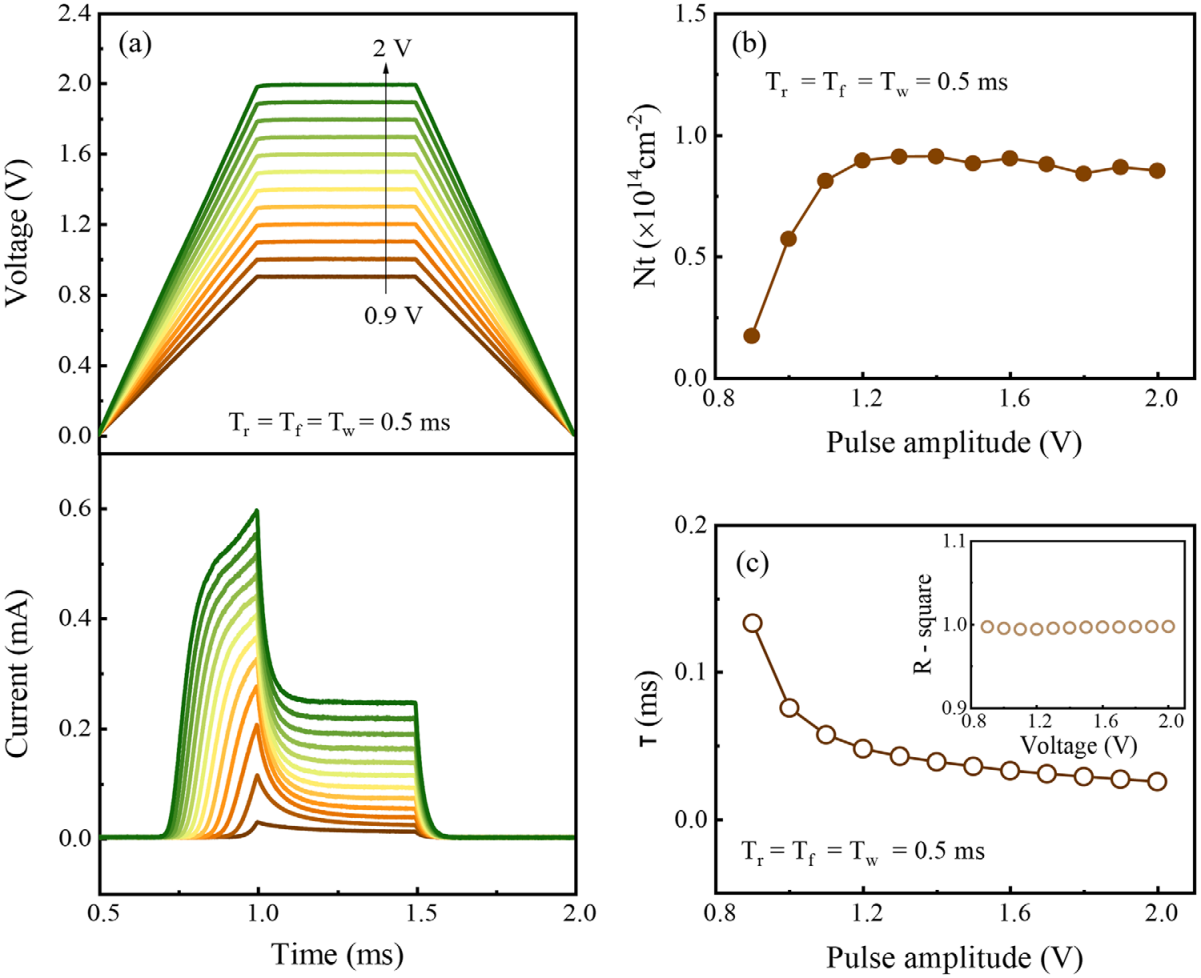
a) (Top) Applied voltage waveforms with pulse amplitudes ranging from 0.9 to 2.0 V under fixed T_r_, T_f_, and T_w_ of 0.5 ms. (Bottom) Corresponding transient current responses, plotted in matching colors. b) Trapped carrier density (N_t_) extracted from the transient current decay during the constant‐voltage phase for different pulse amplitudes. c) Carrier capture time constant (τ) obtained by exponential fitting of the transient current in the constant‐voltage phase. The inset shows the coefficient of determination (R^2^) for each fitting result, confirming high fitting accuracy.

**Figure** **6**a offers the applied voltage waveforms with pulse amplitudes ranging from 0.9 to 2.0 V, while T_r_, T_f_, and T_w_ are maintained at 0.5 ms. The corresponding transient current responses are plotted in the lower panel with matching colors. The transient curves display comparable temporal profiles across different pulse amplitudes, while the peak current at *t* = T_w0_ increases as the voltage pulse amplitude rises. However, the trapped carrier density during the constant‐voltage phase does not increase monotonically with the pulse amplitude. As shown in Figure 6b, N_t_ increases as the voltage pulse amplitude rises from 0.9 to 1.2 V, but saturates for amplitudes above 1.2 V

This behavior can be explained by depletion‐width modulation by the forward bias. During the ramp‐up phase, the depletion width decreases from its equilibrium value to a minimum determined by the maximum forward bias. Traps located in the region that is depleted at equilibrium but neutral at maximum bias contribute to the transient decay observed during the constant‐voltage phase. As the maximum forward bias increases, the depletion width narrows further, engaging more traps in the capture process and thereby increasing N_t_. Once the forward bias exceeds ≈1.2 V, the depletion region nearly vanishes, so the number of contributing traps saturates. Since T_r_ is fixed at 0.5 ms for all cases, the number of traps activated during the ramp‐up phase remains the same, which explains why N_t_ remains nearly constant at higher pulse amplitudes.

Figure 6c presents the carrier capture time constant, τ, extracted from exponential fitting of the transient current during the constant‐voltage phase. The fitting equation is the same as that used in Figure 4c, and the high accuracy of the fitting is confirmed by R^2^ > 0.9999, as shown in the inset. The extracted τ decreases with increasing pulse amplitude. This trend is consistent with the relation τ ∝ (nσv)^−1^, where n is the carrier density, σ is the trap capture cross section, and v is the carrier velocity. At higher forward bias, the increased current corresponds to higher carrier velocity, which leads to shorter capture times.

**Figure 7:**
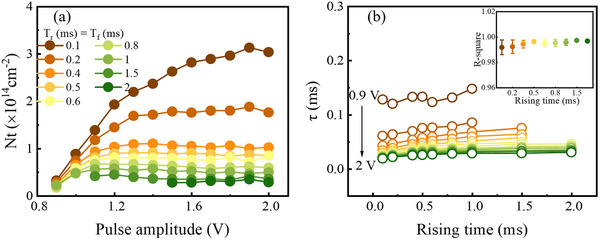
a) Trapped carrier density (N_t_) as a function of pulse amplitude for different rising and falling times (T_r_ = T_f_) ranging from 0.1 to 2.0 ms, with the pulse width fixed at 0.5 ms. b) Carrier capture time constant (τ) extracted from exponential fitting of the transient current decay during the constant‐voltage phase, plotted as a function of T_r_ for pulse amplitudes between 0.9 and 2.0 V. The inset presents the R^2^ of the exponential fits, confirming the reliability of the extracted τ values.

**Figure** **7**a shows the trapped carrier density N_t_ extracted as a function of pulse amplitude for various rising and falling times (T_r_ = T_f_) ranging from 0.1 to 2.0 ms, with the T_w_ fixed at 0.5 ms. Figure S2 (Supporting Information) provides the corresponding transient current responses for each condition. These results are consistent with the trends observed in Figures 4b and 6b. As the forward bias increases, N_t_ rises, reaching a maximum near 1.2 V and then gradually saturates. This behavior indicates that the depletion width approaches its minimum ≈1.2 V and undergoes only minor changes at higher voltages.

Moreover, shorter T_r_ values yield higher N_t_, whereas longer T_r_ values result in progressively lower N_t_. This dependence can result from the occupation of traps during the ramp‐up phase. For short T_r_, the rapid bias increase allows insufficient time for traps to capture carriers, leaving more carriers to be captured by traps during the constant‐voltage phase. In contrast, for long T_r_, a larger fraction of traps is already occupied during the ramp‐up, thereby reducing the density of carriers captured during the constant‐voltage phase.

Figure 7b delivers the carrier capture time constant (τ) as a function of T_r_ for pulse amplitudes between 0.9 and 2.0 V. Exponential fits to the transient current during the constant voltage phase, used to extract τ, are provided in Figure S2 (Supporting Information). The inset confirms the robustness of the fitting, with R^2^ consistently exceeding 0.985 across all conditions. It indicates that the intrinsic carrier capture dynamics of the traps are independent of T_r_ and primarily governed by carrier velocity, which is regulated by forward bias.

**Figure 8:**
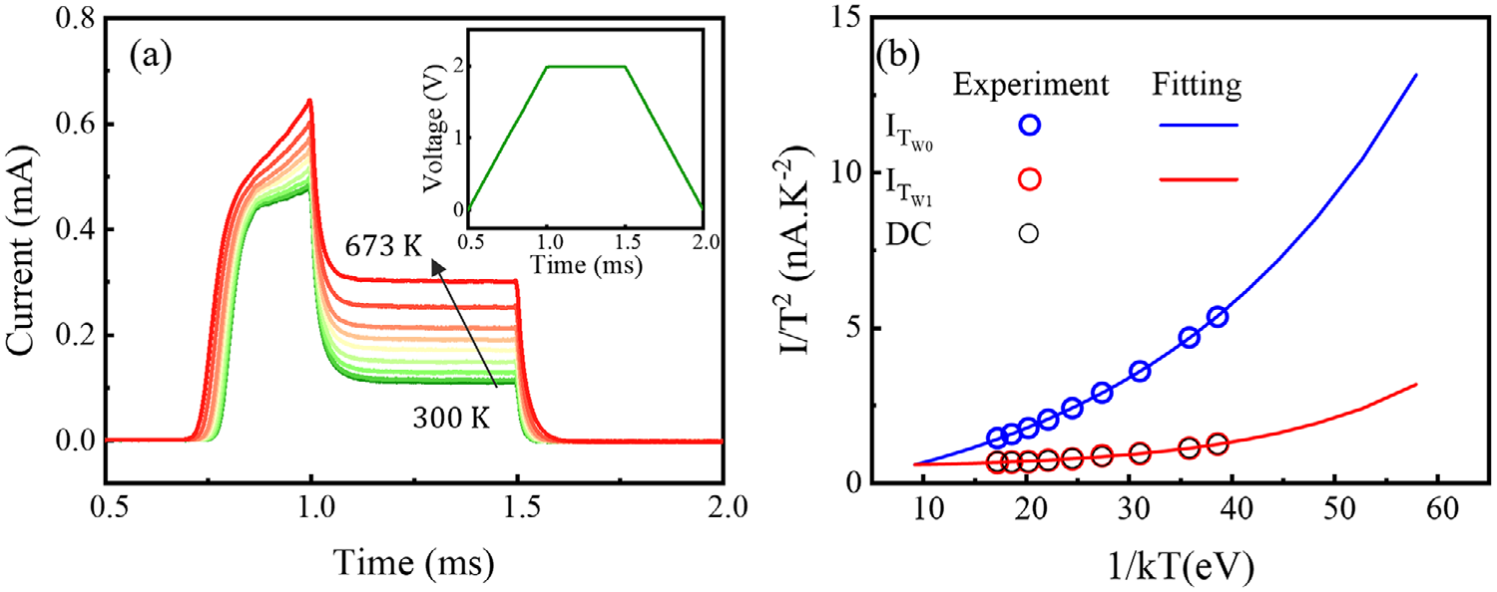
a) Transient current responses of the *β*‐Ga_2_O_3_ SBD measured over a temperature range from 300 to 673 K in 50 K increments. The inset shows the applied voltage waveform, with T_r_, T_f_, and T_w_ fixed at 0.5 ms and an amplitude of 2.0 V. b) Current values I_Tw0_, I_Tw1_, and the DC at V = 2 V plotted in the form of 1/kT versus I/T^2^, where k is the Boltzmann constant. The solid lines represent the results of exponential fitting applied to the experimental data.

**Figure** **8**a presents the transient current responses of the *β*‐Ga_2_O_3_ SBD measured over a temperature range of 300 to 673 K in 50 K increments, under a voltage pulse with T_r_, T_f_, and T_w_ fixed at 0.5 ms and an amplitude of 2.0 V. The inset presents the applied voltage waveform. As the temperature increases, both the peak current at *t* = T_w0_, I_Tw0_, and the current at *t* = T_w1_, I_Tw1_, increase. Additionally, the transient responses during the ramp‐up phase reveal that carrier capture by traps becomes less pronounced at higher temperatures. Between *t* = T_r0_ and *t* = T_r1_, the slope of the initial current rise remains nearly identical across all temperatures. In contrast, in the subsequent interval from *t* = T_r1_ to *t* = T_w0_, where carrier capture by traps strongly influences the current response, the slope of the current rise becomes steeper with increasing temperature. It indicates that carrier capture by traps in *β*‐Ga_2_O_3_ decreases as temperature increases, allowing I_Tw0_ to reach higher values at elevated temperatures.

Figure 8b quantifies I_Tw0_ and I_Tw1_ by plotting the current in the form of 1/kT versus I/T^2^, where k is the Boltzmann constant. For comparison, the current measured under DC conditions at 2 V is also included. The DC data nearly coincide with I_Tw1_ across the entire temperature range, confirming that I_Tw1_ corresponds to the steady‐state current under each condition. The current of a Schottky diode based on the thermionic emission model follows an exponential dependence on temperature:^[^ ^39^, ^40^ ^]^

(2) $$I\;=\;AA^{*}T^{2}\exp\left(-\frac{\Phi_{B}}{\mathrm{kT}}\right)\left\{\exp\left(\frac{qV_{a}}{\mathrm{kT}}\right)-1\right\}$$

where A is the device area, A^⁎^ is the Richardson constant, Φ_B_ is the Schottky barrier height, T is the temperature, q is the electron charge, and V_a_ is the applied voltage, and exp(qV_a_/kT) >> 1 when V_a_ = 2 V. Based on the relation, exponential fitting was performed using the data in Figure 8b. The fitting employed the relation y = A_1_×exp(‐*x*/t_1_) + y_0_, yielding excellent agreement with R^2^ values exceeding 0.992 for all datasets. From the fitting parameter t_1_ of −15.57 obtained from the dataset of I_Tw1_, the Schottky barrier height Φ_B_ was extracted as 1.94 eV, which closely matches the value obtained independently from the C–V analysis shown in Figure S1 (Supporting Information).

Notably, the two exponential curves converge at a single point corresponding to T ≈ 1260 K, where I_Tw0_ equals I_Tw1_. Under this condition, the current decay associated with carrier capture by traps during the constant‐voltage phase no longer occurs, resembling the transient response of the Si SBD shown in Figure 1c. At such elevated temperatures, the average thermal kinetic energy of free electrons can become comparable to or exceed the activation energy of traps within the bandgap of *β*‐Ga_2_O_3_. When this occurs, a significant fraction of electrons can acquire enough energy to escape from these traps, leading to efficient detrapping and a decrease in carrier capture by traps. Therefore, the trap activation energy can be roughly estimated from the average thermal kinetic energy of free electrons, given by E = (3/2)kT, as ≈0.16 eV. This value is consistent with previous reports obtained using DLTS,^[^ ^15^, ^41^, ^42^ ^]^ DLOS,^[^ ^15^, ^43^ ^]^ and thermally stimulated techniques.^[^ ^20^, ^44^, ^45^ ^]^

## 3 Conclusion

This work demonstrates a single‐pulse characterization method for directly probing trap dynamics in the *β*‐Ga_2_O_3_ SBDs. By analyzing transient current responses under varied pulse widths, rise and fall times, amplitudes, and temperatures, we systematically extracted trap parameters and clarified their influence on carrier transport. The results reveal that traps in the neutral region progressively participate in electron capture, leading to current decay during the constant‐voltage phase. This delayed trap response also accounts for the asymmetric behaviors observed during ramp‐up and ramp‐down. The extracted trapped carrier density, ≈5 × 10^14^ cm^−2^, represents the total trap density in the vicinity of the Schottky barrier junction. Furthermore, the carrier capture time constant was determined to be ≈30 µs at a forward bias of 2 V. Temperature‐dependent measurements confirmed the suppression of carrier capture at elevated temperatures and yielded a trap activation energy of ≈0.16 eV, consistent with previously reported values. Overall, the proposed single‐pulse technique offers a straightforward and effective means to evaluate trap states under conditions directly relevant to device operation, thereby enhancing the understanding of defect‐related effects in *β*‐Ga_2_O_3_ devices.

## 4 Experimental Section

### Device Fabrication

An 11‐µm‐thick Si‐doped n‐type *β*‐Ga_2_O_3_ epitaxial layer was grown on a (001)‐oriented Sn‐doped n‐type *β*‐Ga_2_O_3_ single‐crystal wafer by hydride vapor phase epitaxy (HVPE). The epitaxial wafers were purchased from Novel Crystal Technology (Japan). The epitaxial layer exhibited a carrier concentration of 1.9×10^16^ cm^−3^, while the bulk substrate, serving as the conductive back contact, had a higher carrier concentration of 5.0×10^18^ cm^−3^.

Ohmic contact formation was achieved by depositing a Ti/Au (20/100 nm) metal stack on the backside of the substrate via electron beam evaporation, followed by rapid thermal annealing (RTA) at 500 °C for 60 s in a nitrogen (N_2_) ambient. Schottky contacts were subsequently fabricated on the front surface of the *β*‐Ga_2_O_3_ epitaxial layer. Circular patterns with a diameter of 320 µm were defined using photolithography, and a Pt/Ti/Au (20/10/100 nm) metal stack was deposited by electron beam evaporation, followed by metal lift‐off.

### Electrical Characterizations

All the characteristics of the SBD were measured using a semiconductor parameter analyzer (Keithley 4200 SCS). The device was mounted on a ceramic heater within a sealed Nextron Micro Probe System (Republic of Korea) chamber for temperature‐dependent measurements. The temperature was precisely controlled from 300 to 673 K (±1 K) using a Nextron temperature controller. A HiCube 300H Neo Pfeiffer vacuum system was used to minimize background contamination, maintaining pressures in the range of 10^−4^ to 10^−5^ hPa.

For direct current (DC) current‐voltage (*I–V*) characteristics were measured by performing a double voltage sweep (hysteresis measurement) from 0 to 2 V and back to 0 V, with a voltage step of 25 mV. The capacitance–voltage (C–V) characteristics of the SBD, given in Figure S1 (Supporting Information), indicate that the doping concentration of the *β*‐Ga_2_O_3_ epitaxial layer was 1.3×10^16^ cm^−3^ and the built‐in potential of the Schottky barrier was 1.82 V. The doping concentration was in good agreement with the value obtained following epitaxial growth. The Schottky barrier height of the fabricated Pt/*β*‐Ga_2_O_3_ SBD, extracted from the built‐in potential, was ≈1.98 eV, consistent with previously reported values, confirming the reliability of the devices.^[^ ^46^ ^]^

For pulse response characterization, two 4225‐PMU modules connected to 4225‐RPM switching units were employed, enabling current sensitivity down to the nA range and automated switching between DC and pulsed current (PC) measurement modes. A trapezoidal voltage pulse was applied, and the transient current response of the SBD was recorded. To systematically probe the trap dynamics, the pulse parameters were varied across different experiments: the pulse amplitude was swept from 0.9 to 2.0 V, the pulse width (T_w_) was varied from 0.05 to 4 ms, and the rising (T_r_) and falling (T_f_) times were independently adjusted from 0.05 to 2.5 ms.

## Conflict of Interest

The authors declare no conflict of interest.

## Supporting information



Supporting Information

## Data Availability

The data that support the findings of this study are available from the corresponding author upon reasonable request.
