# Supplementary material for: Direct Probing of Trap Dynamics in β‐Ga2O3 Schottky Barrier Diodes Using Single‐Voltage‐Pulse Characterization
Source: Adv Sci (Weinh). 2025 Dec 14;13(13):e18859. doi: 10.1002/advs.202518859 (PMC12955861; doi:10.1002/advs.202518859)
Supplement: Supplementary file 1 — Supporting Information [file ADVS-13-e18859-s001.docx]

Supporting Information

Direct Probing of Trap Dynamics in *β*-Ga_2_O_3_ Schottky Barrier Diodes Using Single-Voltage-Pulse Characterization

Thanh Huong Vo, Sunjae Kim, Ji-Hyeon Park, Dae-Woo Jeon, Wan Sik Hwang, and Jinyoung Hwang *


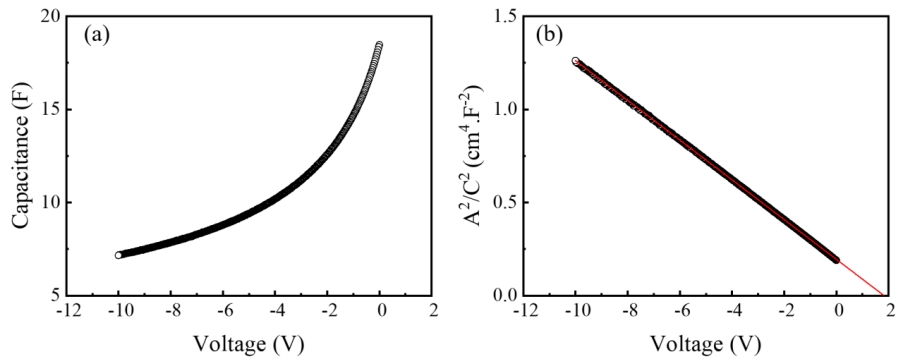


**Figure S1**. (a) Capacitance – voltage with frequency 1MHz. (b) A^2^/C^2^ and the fitting line to obtain built in potential (V_bi_ = 1.82 V) via the intercept and doping concentration
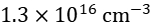
 by the slope.


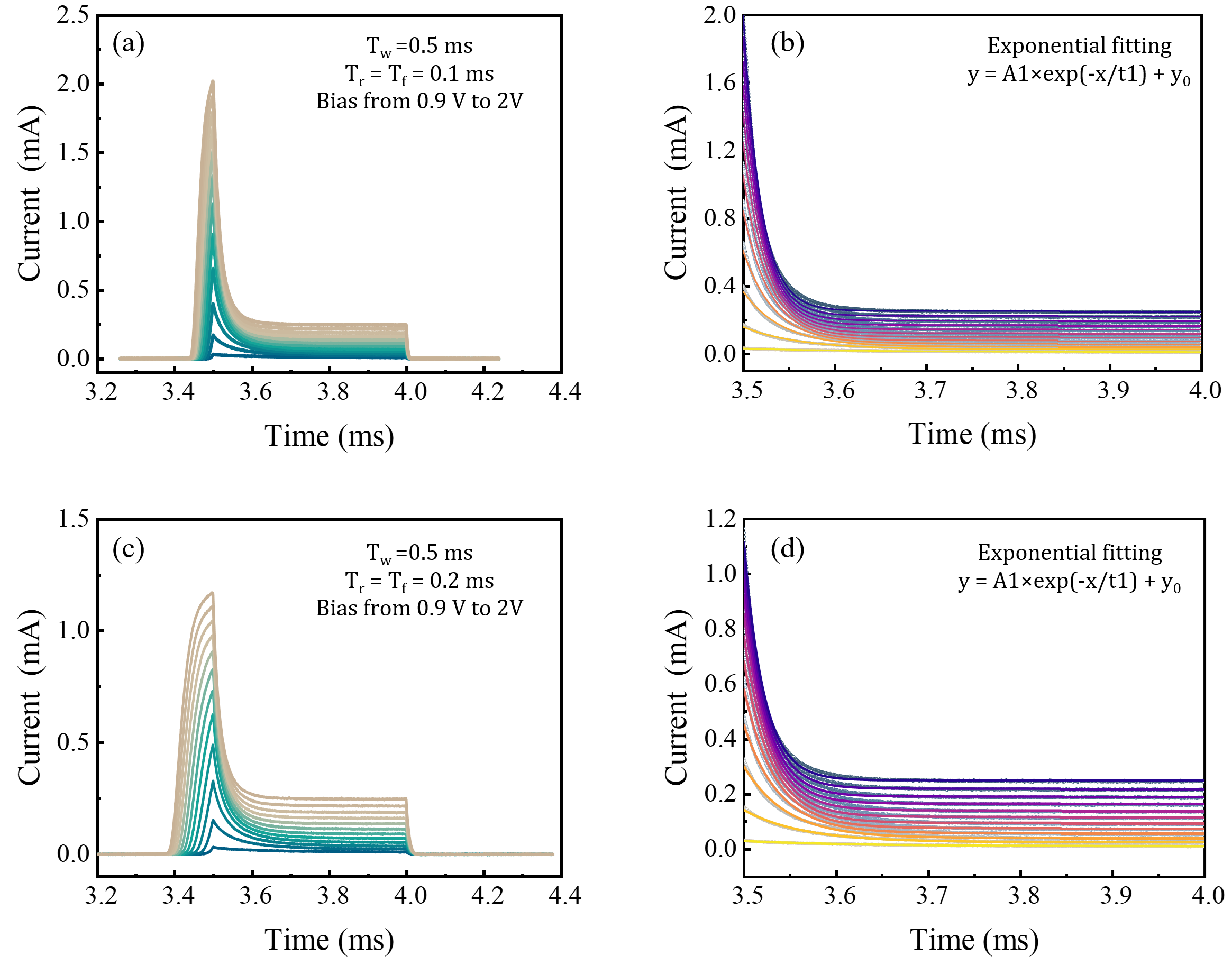


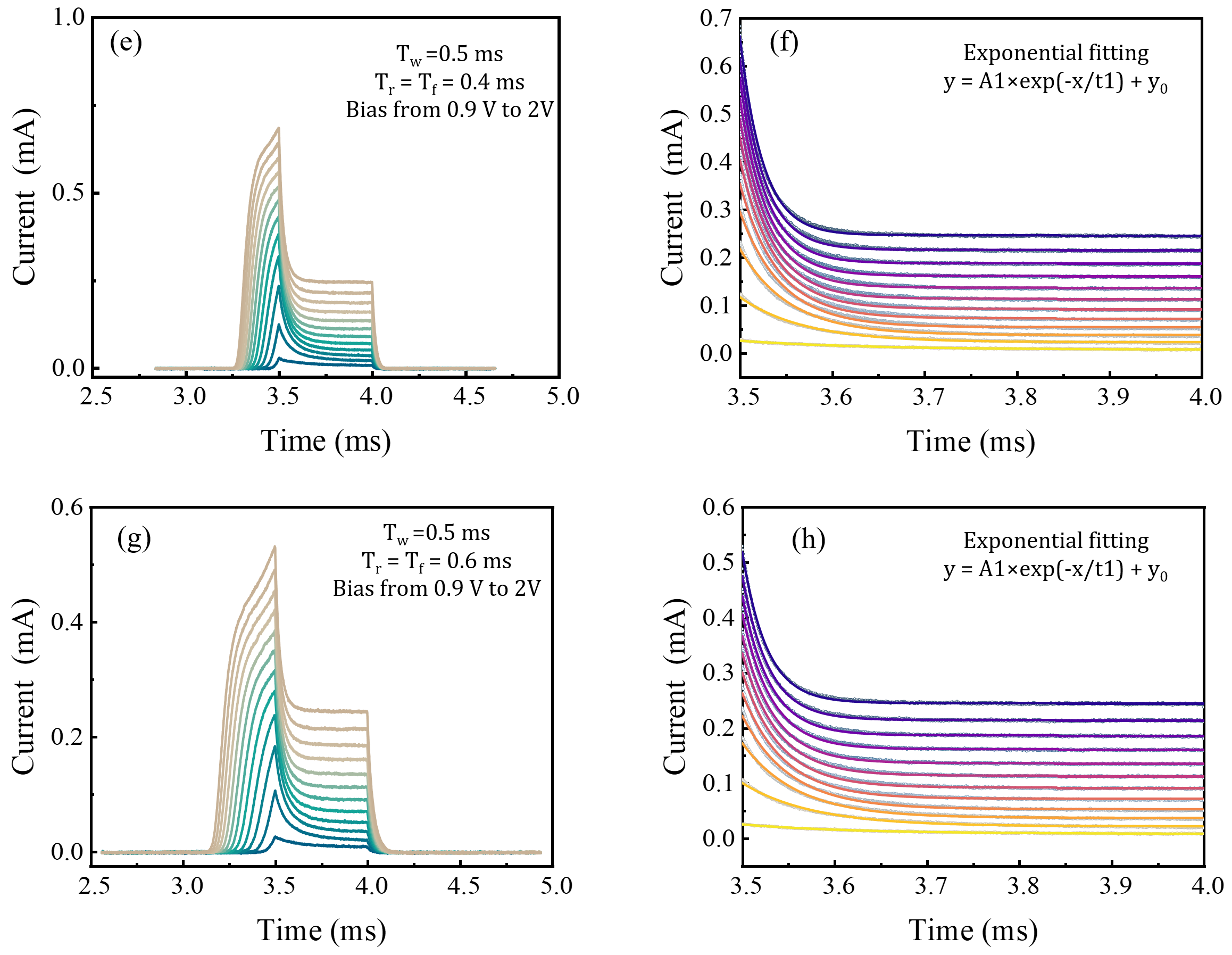


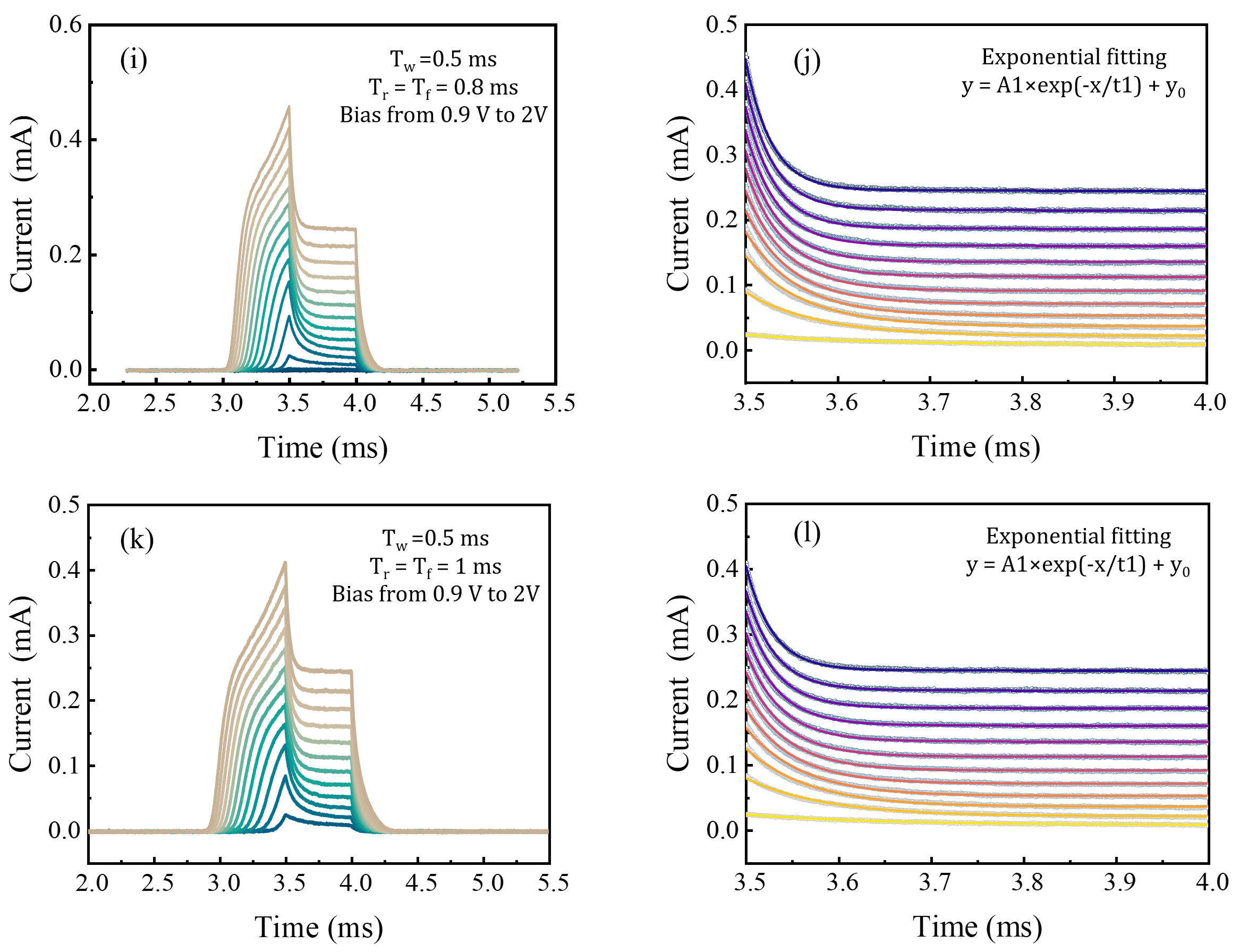


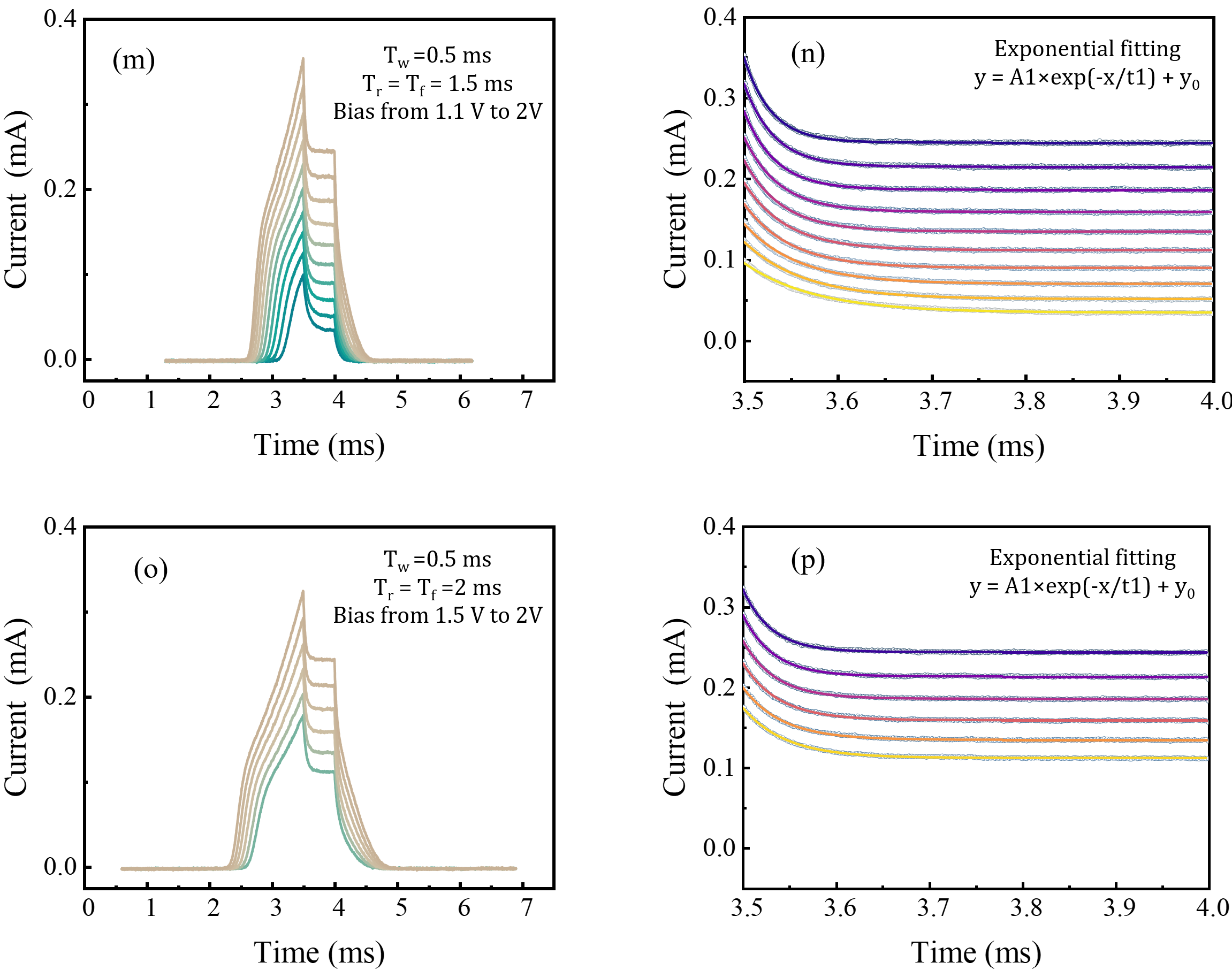


**Figure S2**. Single pulse current with time width T_w_=0.5ms, pulse amplitude 0.9 V to 2 V, and rising time, falling time T_r_ = T_f_ (left), and the exponential fitting (right) alternated from (a, b) 0.1 ms; (c, d) 0.2 ms; (e, f) 0.4 ms; (g, h) 0.6 ms; (i, j) 0.8 ms; (k, l) 1 ms; (m, n) 1.5 ms; (o, p) 2 ms.
